# Irisin reverses intestinal epithelial barrier dysfunction during intestinal injury via binding to the integrin αVβ5 receptor

**DOI:** 10.1111/jcmm.14811

**Published:** 2019-11-07

**Authors:** Jianbin Bi, Jia Zhang, Yifan Ren, Zhaoqing Du, Teng Li, Tao Wang, Lin Zhang, Mengzhou Wang, Zheng Wu, Yi Lv, Rongqian Wu

**Affiliations:** ^1^ National Local Joint Engineering Research Center for Precision Surgery & Regenerative Medicine Shaanxi Provincial Center for Regenerative Medicine and Surgical Engineering Institute of Advanced Surgical Technology and Engineering First Affiliated Hospital of Xi’an Jiaotong University Xi’an China; ^2^ Department of Hepatobiliary Surgery First Affiliated Hospital of Xi’an Jiaotong University Xi’an China

**Keywords:** AMP‐activated protein kinase, enterocytes, gut barrier function, integrin αVβ5, UCP 2

## Abstract

Disruption of the gut barrier results in severe clinical outcomes with no specific treatment. Metabolic disorders and destruction of enterocytes play key roles in gut barrier dysfunction. Irisin is a newly identified exercise hormone that regulates energy metabolism. However, the effect of irisin on gut barrier function remains unknown. The therapeutic effect of irisin on gut barrier dysfunction was evaluated in gut ischemia reperfusion (IR). The direct effect of irisin on gut barrier function was studied in Caco‐2 cells. Here, we discovered that serum and gut irisin levels were decreased during gut IR and that treatment with exogenous irisin restored gut barrier function after gut IR in mice. Meanwhile, irisin decreased oxidative stress, calcium influx and endoplasmic reticulum (ER) stress after gut IR. Moreover, irisin protected mitochondrial function and reduced enterocyte apoptosis. The neutralizing antibody against irisin significantly aggravated gut injury, oxidative stress and enterocyte apoptosis after gut IR. Further studies revealed that irisin activated the AMPK‐UCP 2 pathway via binding to the integrin αVβ5 receptor. Inhibition of integrin αVβ5, AMPK or UCP 2 abolished the protective role of irisin in gut barrier function. In conclusion, exogenous irisin restores gut barrier function after gut IR via the integrin αVβ5‐AMPK‐UCP 2 pathway.

## 1 INTRODUCTION

The intact intestinal epithelial monolayer provides an essential structural basis for intestinal absorption and serves as a permeability barrier.^1^ Gut ischemia reperfusion (IR) injury, occurring in clinical conditions such as superior mesenteric artery occlusion and intestinal transplantation, severely destroys intestinal barrier functions.^2^ Consequently, some intraluminal microorganisms and endotoxin enter the blood and other organs, further resulting in severe clinical outcomes.^3^ Effective methods for the prevention and treatment of intestinal barrier injury are urgently needed.

An increasing number of evidence manifest reactive oxygen species (ROS) plays a pivotal role in the pathogenesis of gut IR.^4^ On one hand, excessive accumulation of ROS gives rise to calcium influx and endoplasmic reticulum (ER) stress resulting in mitochondrial dysfunction, disrupted intercellular tight junctions and enterocyte apoptosis.^5^ On the other hand, ROS accelerates inflammatory cell infiltration and cytokine release, which further fuel ROS generation as a positive feedback during gut IR.^6^ Massive enterocyte apoptosis and disrupted intercellular tight junctions are the main mechanisms of intestinal barrier dysfunction.^7^ As a protective mechanism, mitochondrial uncoupling protein 2 (UCP 2) serves as a negative feedback regulator in the presence of excessive ROS. Overexpression of UCP 2 significantly decreased oxidative stress and cell apoptosis.^8^ Accumulating evidence suggests that AMP‐activated protein kinase (AMPK) plays a pivotal role in gut barrier function.^9^ As an energy sensor, AMPK regulates mitochondrial function and the energy requirement of enterocytes.^10^, ^11^, ^12^ Besides, AMPK can directly facilitate the aggregation of cytoskeletal proteins and formation of intercellular tight junctions via activation of Rac1.^9^, ^13^

Since first reported in 2012, irisin has been an intriguing option for solving obesity problems.^14^ As a newly identified hormone, the major function of irisin is regulating glucose/lipid metabolism and mitochondrial function.^14^, ^15^ However, as research continues, it has been shown that irisin also benefits type 2 diabetes, ageing and some cardiovascular diseases.^16^, ^17^, ^18^ In addition, many studies have shown that irisin can facilitate AMPK activation to further regulate energy metabolism.^13^ A recent study indicated that irisin restrains bone loss by binding to the αv class of integrins in osteocytes.^19^ However, the effect of exogenous irisin on gut barrier function has not been elucidated to date. We therefore suggested that irisin restores gut barrier function after gut IR via activation of the integrin αvβ5‐AMPK‐UCP 2 pathway. The main purpose of this study was to determine the effects of exogenous irisin on gut barrier function after gut IR. In addition, our study also sought to clarify the effects of irisin on the integrin αvβ5‐AMPK‐UCP 2 pathway during gut IR injury.

## 2 MATERIALS AND METHODS

### 2.1 Experimental animals

Experiments were performed on male wild‐type C57BL/6 J mice (aged 6‐8 weeks, weighing 22‐25 g, Experimental Animal Center of Xi'an Jiaotong University). Mice were anaesthetized with isoflurane gas. The protocol was developed according to the guidelines of the China Council on Animal Care and Use and approved by the Institutional Animal Care and Use Committee of the Ethics Committee of Xi'an Jiaotong University Health Science Center, China (approval number: 2017‐564).

### 2.2 Mouse model of gut IR

A mouse model of gut IR was conducted as described previously.^20^ The superior mesenteric artery (SMA) was occluded with an atraumatic clip for 60 minutes, and then, reperfusion was allowed under anaesthesia with isoflurane. Sham group mice were given 0.5 mL saline after sham operation without ischemia treatment; mice were intravenously administered 250 μg/kg irisin (067‐29A; Phoenix Pharmaceuticals, Inc), 20 mg/kg cilengitide trifluoroacetate (S707; Selleck) or 20 mg/kg genipin (S2412; Selleck, China) immediately after reperfusion. Four hours later, the mice were euthanized, and the following experiments were performed. In additional groups of animals, anti‐irisin (4 mg/kg; Phoenix Pharmaceuticals) blocking antibody was administered at 24 hours before gut IR.

### 2.3 Cell culture, hypoxia/reoxygenation, irisin, cilengitide and genipin treatment

Caco‐2 cells (human colon carcinoma, CL‐0050; Procell Life Science & Technology) were cultured in MEM medium (A1049001; Gibco) supplemented with 20% foetal bovine serum (FBS. 10 099 141; Gibco) and 100 units/mL penicillin/streptomycin mixture (15 070 063; Gibco). Cells were incubated at 37°C with 100% humidity and 5% CO_2_. To induce the hypoxia/reoxygenation (H/R) cell culture model, Caco‐2 cells were cultured in MEM medium (glucose/FBS free) and exposed to hypoxia conditions (94% N_2_, 5% CO_2_ and 1% O_2_) at 37°C for 90 minutes. Sham group cells were given equivoluminal PBS without H/R treatment; the Caco‐2 cells were administered with 10 nmol/L irisin, 20 μmol/L cilengitide trifluoroacetate or 20 μmol/L genipin immediately after reperfusion.

### 2.4 Depletion of AMPK

The siRNA transfection experiment was performed as described previously.^21^ AMPK‐specific small interfering RNA (siRNA): 5′‐CGGGAUCAGUUAGCAACUATT‐3′ and 5′‐UAGUUGCUAACUGAUCCCGTT‐3′ and nonspecific siRNA (GenePharma) were used to transfect Caco‐2 cells.

### 2.5 Histological analysis and gut IR score

Haematoxylin and eosin staining of fixed intestinal tissues was performed as described previously.^22^ Images were collected by a light microscope, and a representative field was chosen for assessment. The gut IR score was graded as follows: 0, normal mucosal villi; 1, minor subepithelial space and capillary congestion; 2, extensive subepithelial space with little epithelial layer lifting from the lamina propria; 3, massive epithelial layer lifting from the lamina propria; and 4, villi detachment and haemorrhage.^23^

### 2.6 Water content

Gut tissues were weighed immediately (wet weight) and at 48 hours after drying in a 60°C oven (dry weight). Gut water content was calculated as H_2_O % = (1 − dry weight/wet weight) × 100%.

### 2.7 FITC‐dextran permeability assay

Mice were administered 200 μL FITC‐dextran (25 mg/mL) immediately after reperfusion by gavage. Four hours later, mice were sacrificed, and blood FITC‐dextran concentrations were assessed with a Varioskan™ LUX multimode microplate reader (Thermo Scientific™) at an excitation wavelength of 485 nm and an emission wavelength of 515 nm.

### 2.8 Bacterial content

The mesenteric lymph node complex and lung tissues were harvested after euthanasia. The tissues were homogenized and centrifuged to obtain a supernatant. After serial log dilutions, 500 μL each dilution was evenly coated onto chocolate agar plates. The plates were incubated at 37°C for 24 hours, and colony‐forming units (CFUs) were counted.

### 2.9 Enzyme‐linked immunosorbent assays

Serum irisin TNF‐α and CIRP were determined with an irisin enzyme‐linked immunosorbent assays (ELISA) kit (EK‐067‐29; Phoenix Pharmaceuticals, Inc), a TNF‐α mouse ELISA kit (BMS607‐3; Thermo Fisher Scientific) and a CIRP ELISA kit (CSB‐EL005440MO; Cusabio) following the manufacturer's instructions.

### 2.10 Measurement of oxidative stress

Levels of gut malonaldehyde (MDA), xanthine oxidase (XO), 4‐hydroxynonenal (4‐HNT), superoxide dismutase (SOD) and glutathione peroxidase activity (GSH‐PX) were detected by MDA assay Kit (A003‐1), XO assay Kit (A002‐1‐1), 4‐HNT assay Kit (H268), SOD assay Kit (A001‐3) and GSH‐PX assay Kit (A005) purchased from NanJing JianCheng Bioengineering Institute, according to the instructions of the manufacturer.

### 2.11 Western blot analysis

Western blot analysis was performed as described previously.^22^ PVDF membranes were incubated with primary rabbit anti‐irisin antibody (1:1000 dilution, ab174833; Abcam); anti‐claudin‐1 antibody (1:1000 dilution, 13050‐1‐AP; Proteintech); rabbit anti‐occludin antibody (1:1000 dilution, ab216327; Abcam); rabbit anti‐AMPKα antibody (1:1000 dilution; Cell Signaling Technology); rabbit anti‐PAMPKα antibody (1:1000 dilution; Cell Signaling Technology); rabbit anti‐UCP 2 antibody (1:1000 dilution, ab203244; Abcam); rabbit anti‐UCP1 antibody (1:1000 dilution, 14670S; Cell Signaling Technology); rabbit anti‐IRE1 antibody (1:1000 dilution, ab37073; Abcam); rabbit anti‐CHOP antibody (1:1000 dilution, 5554; Cell Signaling Technology); or mouse anti‐β‐actin monoclonal antibody (1:1000 dilution, HRP‐60008; Proteintech) overnight at 4°C. Then, secondary HRP‐conjugated goat anti‐rabbit IgG (1:2000 dilution, SA00001‐2; Proteintech) was incubated for 1 hour at room temperature. Protein expression was detected by a chemiluminescence system (Bio‐Rad) and quantified by ImageJ2x software.

### 2.12 Coimmunoprecipitation

Coimmunoprecipitation (CO‐IP) was conducted as described previously.^24^ Protein A/G PLUS‐Agarose (sc‐2003; Santa) and mouse anti‐integrin alpha V/beta 5 antibody (sc‐81632; Santa) were used for immunoprecipitation. Rabbit anti‐irisin antibody (1:1000, ab174833; Abcam), rabbit anti‐integrin alpha V antibody (1:1000, ab179475; Abcam) and rabbit anti‐integrin beta 5 antibody (1:1000, ab15459; Abcam) were used for WB.

### 2.13 Activation of Rac1

Activated and total Rac1 levels were determined with a Rac1 Activation Assay Combo Kit (STA‐405; Cell Biolabs) following the manufacturer's instructions.

### 2.14 Immunofluorescence

Immunofluorescence staining was performed as described previously.^22^ Samples were incubated with rabbit anti‐irisin antibody (1:200 dilution, NBP2‐59680; Novus), rabbit anti‐JAM‐A antibody (1:100 dilution, ab180821; Abcam), rabbit anti‐ZO‐1 antibody (1:100 dilution, ab96587; Abcam) and mouse anti‐integrin alpha V/ beta 5 antibody (1:100 dilution, sc‐81632; Santa). A confocal microscope (TCS SP8 STED 3X; Leica) was used for image capture.

### 2.15 Measurement of TER

The TER of Caco‐2 cells was determined by an electrical cell‐substrate impedance sensing system (Applied Biophysics) as described previously.^25^

### 2.16 Transwell permeability assays

Transwell permeability assays were performed using 6.5‐mm transwell dishes with 0.4 µm pore polycarbonate membrane inserts (3413; Corning). FITC‐albumin concentrations were assessed with a Varioskan™ LUX multimode microplate reader (Thermo Scientific™) at an excitation wavelength of 485 nm and an emission wavelength of 515 nm.

### 2.17 TUNEL, MitoTracker Red, DHE and Fluo‐4 AM fluorescence staining

A TUNEL kit (11 684 795 910; Roche) was used for TUNEL staining according to the manufacturer's instructions. Two hundred nanomolar MitoTracker Red CMXRos dye (M7512; Thermo Fisher Scientific), 3 μmol/L dihydroethidium (DHE) dye (D7008; Sigma‐Aldrich) and 2 μmol/L Fluo‐4 AM (S1060; Beyotime) were incubated for 20, 30 and 25 minutes to detect mitochondria, ROS and Ca^2+^, respectively. The stained cells were observed with a confocal microscope (TCS SP8 STED 3X; Leica).

### 2.18 Flow cytometry analysis

An annexin V‐FITC/PI Apoptosis Detection Kit (AD10; Dojindo Laboratories) was used to detect Caco‐2 cell apoptosis with flow cytometry (ACEA Biosciences, Inc) according to the manufacturer's instructions. The percentage of apoptotic cells was calculated from the sum of early apoptosis and late apoptosis (n = 3 per group).

### 2.19 Determination of ATP content

ATP content was determined by an ATP Assay Kit (S0026; Beyotime Biotechnology) according to the manufacturer's instructions.

### 2.20 Analysis of mitochondrial DNA content

Mitochondrial DNA (mtDNA) content was detected as mtDNA encoded NADH dehydrogenase‐1 and normalized against the nuclear encoded POU class 5 homeobox 1 gene as described previously.^22^

### 2.21 Determination of LDH levels

Serum LDH was quantified by using an assay kit (A020‐2; Nanjing Jiancheng Bioengineering Institute) according to the manufacturer's instructions.

### 2.22 Statistical analysis

Results were expressed as the means ± standard error of the mean (SEM). *t* Test or one‐way ANOVA was applied to analyse the differences between groups by SPSS 18.0. *P* < .05 represents a significant difference.

## 3 RESULTS

### 3.1 Exogenous irisin restores gut barrier function after gut IR

**Figure 1:**
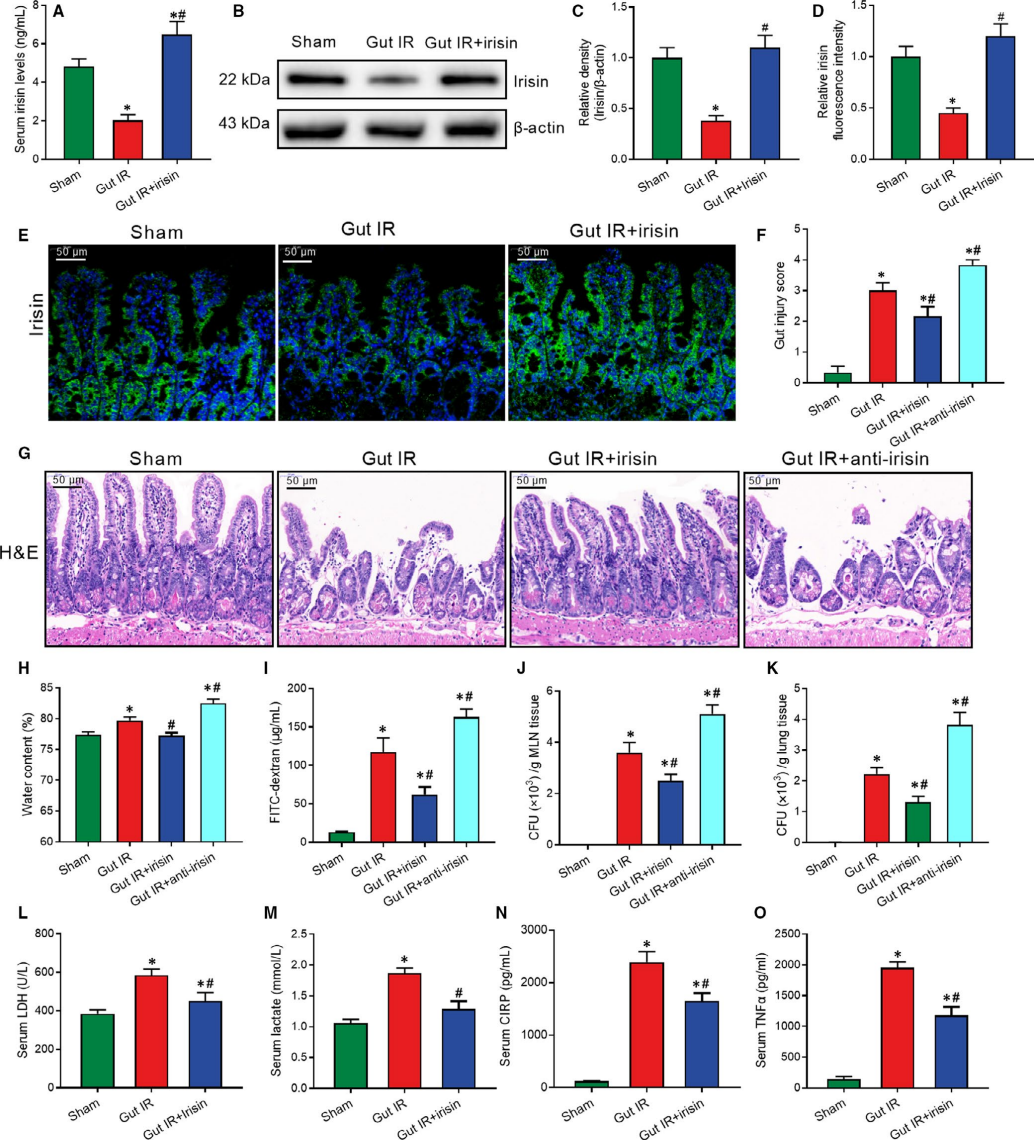
Exogenous irisin restores gut barrier function after gut IR. Irisin (250 μg/kg in 0.5 mL saline, a single dose, iv) was administered immediately after reperfusion. Anti‐irisin (4 mg/kg, Abcam) blocking antibodies were administered at 24 h before gut IR. Four hours after reperfusion, mice were sacrificed, and tissue samples were collected. A, Serum irisin levels; (B,C) Western blot analysis of irisin expression; (D,E) immunofluorescence staining of irisin (green) and the corresponding nuclear counterstaining (blue) in gut tissues; (F) gut injury score; (G) haematoxylin and eosin (H&E) staining; (H) water content of gut; (I) serum FITC‐dextran levels; (J,K) colony‐forming units (CFUs) from mesenteric lymph node (MLN) and lung tissues; (L,M) serum levels of LDH and lactate; and (N,O) serum TNF‐α and CIRP levels. n = 6 per group, mean ± SEM, *P < .05 vs the sham group, ^#^ *P* < .05 vs the gut IR group

A significant reduction in serum irisin was observed after gut IR treatment, while mice received recombinant irisin treatment (250 μg/kg, iv) showed higher irisin levels at 4 hours after gut IR (Figure 1A). Irisin levels in the intestine were detected by Western blot as shown in Figure 1B,C. Mice that underwent gut IR showed a significant reduction in irisin levels and irisin treatment increased the irisin levels in the intestine Immunofluorescence staining showed irisin widely distributed around the intestinal epithelial cells (Figure 1D,E). Histological analysis revealed extensive villi detachment, epithelial necrosis, lamina propria damage and haemorrhage after gut IR, while exogenous irisin‐treated mice showed minor histological changes (Figure 1F,G). Meanwhile, irisin‐treated mice exhibited lower water content than the control‐treated mice after gut IR (Figure 1H). Consistent with the histological changes, a significant increase in serum FITC‐dextran was detected after gut IR, while irisin treatment significantly reversed this change (Figure 1I). Mesenteric lymph node (MLN) and lung bacterial loads were determined, and the results showed that irisin treatment significantly reduced the increase of bacterial translocation to the MLN and lung that occurred after gut IR (Figure 1J,K). Additionally, the neutralizing antibody against irisin significantly aggravated gut injury and increased the levels of water content, serum FITC‐dextran and bacterial loads in gut IR mice (Figure 1F‐K) In addition, irisin treatment markedly decreased the levels of serum LDH and lactate (Figure 1L,M). Moreover, the irisin‐treated group showed lower levels of serum tumour necrosis factor α (TNF‐α) and cold‐inducible RNA binding protein (CIRP) than the control‐treated group (Figure 1N,O).

### 3.2 Irisin increases the intercellular tight junctions between enterocytes after gut IR

**Figure 2:**
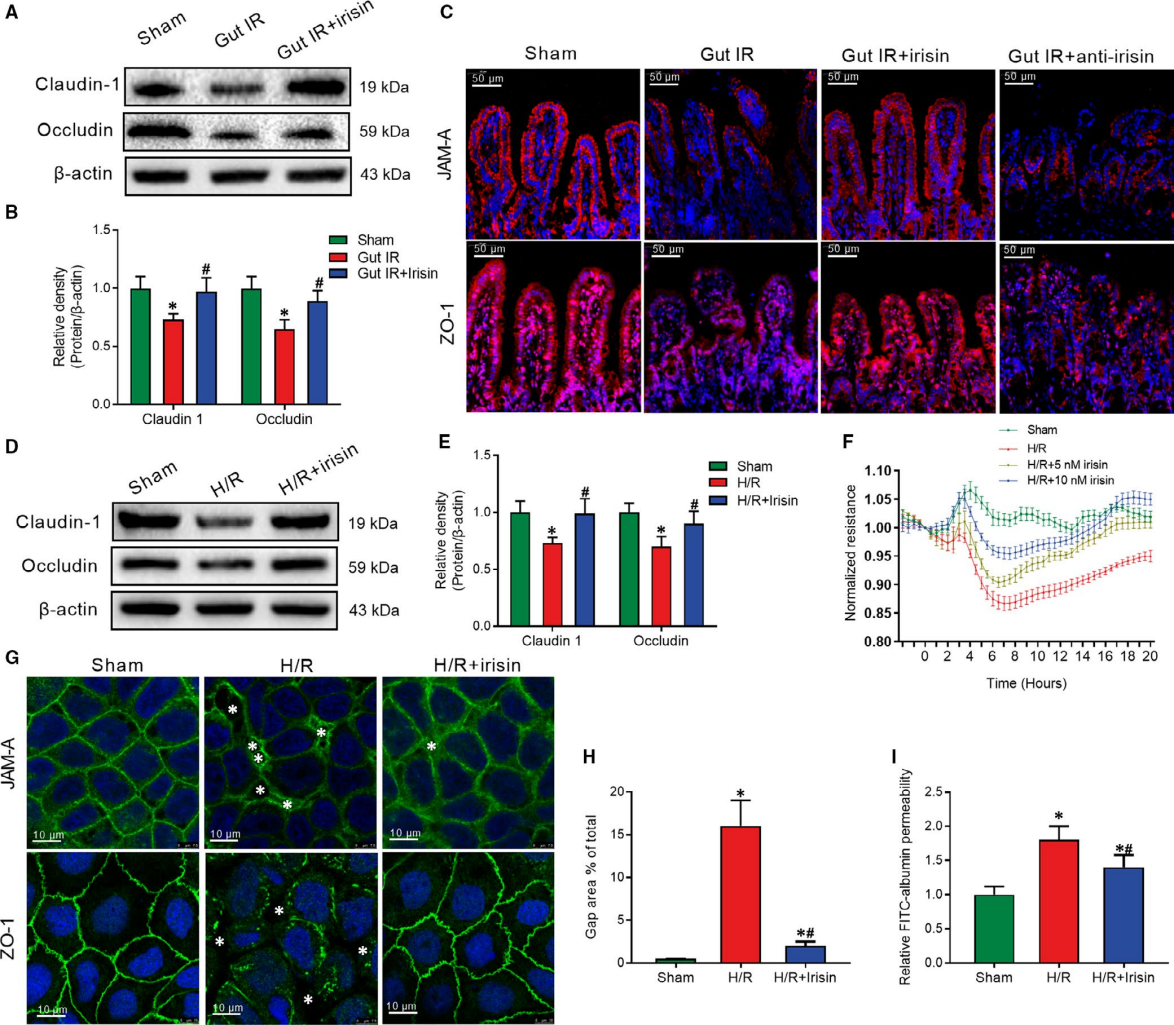
Irisin increases intercellular tight junctions between enterocytes after gut IR. Irisin (250 μg/kg in 0.5 mL saline, a single dose, iv) was administered immediately after reperfusion. Anti‐irisin (4 mg/kg; Abcam) blocking antibodies were administered at 24 h before gut IR. Four hours after reperfusion, mice were sacrificed, and tissue samples were collected. A,B, Western blot analysis of claudin‐1 and occludin expression; (C) immunofluorescence staining of JAM‐1 and ZO‐1 (red) and the corresponding nuclear counterstaining (blue) in gut tissues. n = 6 per group, mean ± SEM, *P < .05 vs the sham group, ^#^ *P* < .05 vs the gut IR group. Caco‐2 cells were exposed to hypoxia for 90 min, and 10 nmol/L irisin was added at the beginning of reoxygenation. (D,E) Western blot analysis of claudin‐1 and occludin expression; (F) transepithelial electrical resistance (TER); (G) immunofluorescence staining of JAM‐1 and ZO‐1 and the corresponding nuclear counterstaining (blue). * in the figure represents intercellular gap; (H) gap area percentage; and (I) relative diffusion of FITC‐labelled albumin at 4 h after reoxygenation in Caco‐2 cells. n = 3 per group, mean ± SEM, *P < .05 vs the sham group, ^#^ *P* < .05 vs the H/R group

Western blot revealed a conspicuous decrease in tight junction‐related claudin‐1 and occludin expression during gut IR injury, but these changes were reversed by exogenous irisin treatment (Figure 2A,B). Immunofluorescent staining showed that irisin treatment increased junctional adhesion molecule‐A (JAM‐A) and ZO‐1 expression and decreased interruption of enterocyte distribution, while the neutralizing antibody against irisin significantly reduced the JAM‐A and ZO‐1 expression after gut IR (Figure 2C). Caco‐2 cells are widely used to simulate the barrier function of enterocytes.^26^, ^27^ Similar to the in vivo results that irisin administration reversed the losses of claudin‐1 and occludin that occurred after hypoxia and reoxygenation (H/R) treatment of Caco‐2 cells (Figure 2D,E). Additionally, massive disruption of the intercellular tight junctions and significant increases in gap areas were observed after H/R treatment. Irisin administration dramatically reversed the changes induced by H/R treatment (Figure 2F,G). In addition, H/R treatment resulted in a marked reduction of transepithelial electrical resistance (TER) of Caco‐2 cells. Treatment with 10 nmol/L irisin alleviated the decreasing trend in TER after H/R treatment, but treatment with 5 nmol/L irisin showed a weaker effect (Figure 2H). Moreover, transwell permeability assays revealed that irisin administration decreased the high permeability of FITC‐albumin after H/R treatment of Caco‐2 cells (Figure 2I).

### 3.3 Irisin decreases the oxidative stress, calcium influx and ER stress after gut IR

**Figure 3:**
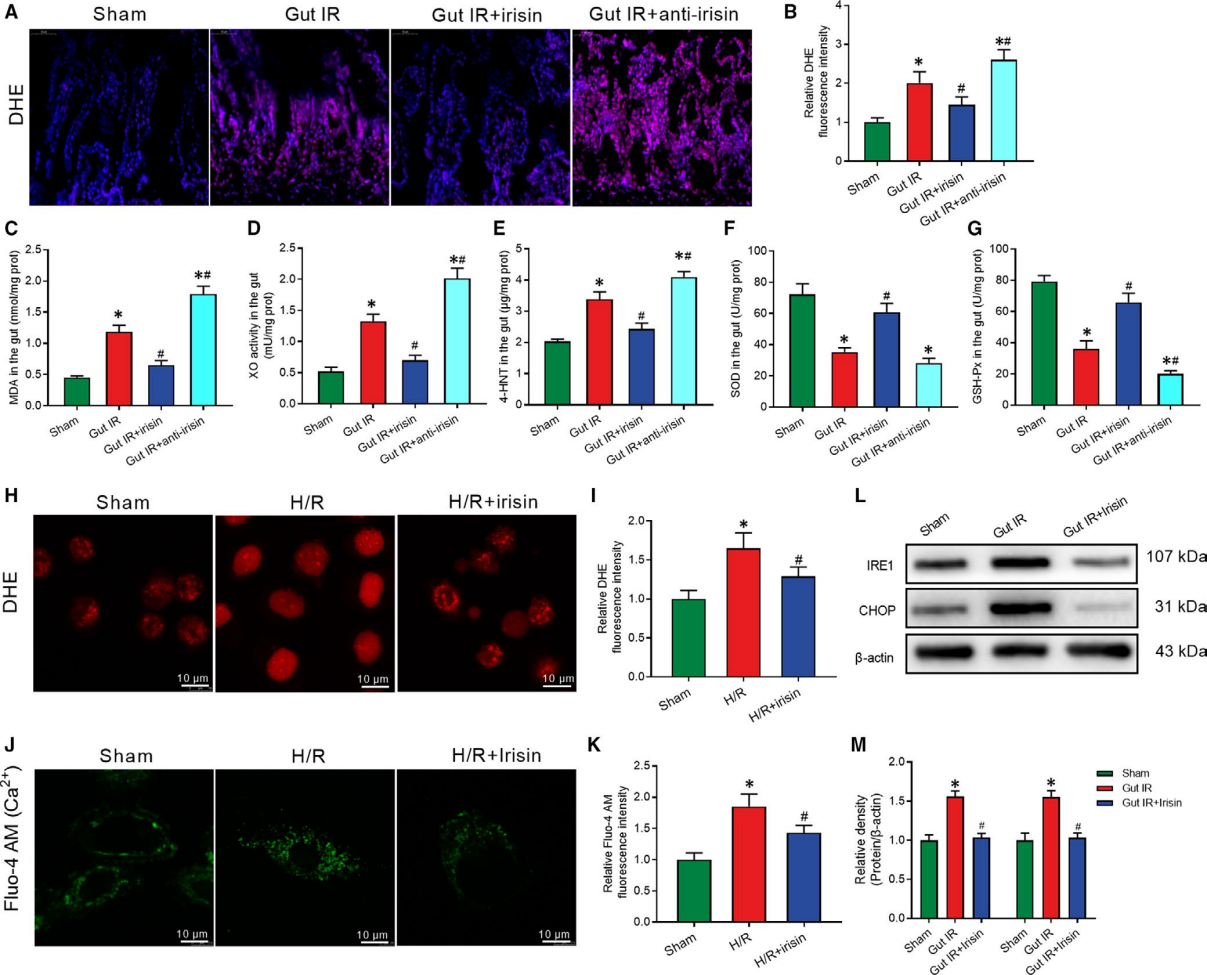
Irisin decreases the oxidative stress, calcium influx and ER stress after gut IR. Irisin (250 μg/kg in 0.5 mL saline, a single dose, iv) was administered immediately after reperfusion. Anti‐irisin (4 mg/kg, Abcam, USA) blocking antibody was administered at 24 h before gut IR. Four hours after reperfusion, mice were sacrificed, and tissue samples were collected. A,B, DHE fluorescence staining of gut tissues; (C‐G) levels of gut malonaldehyde (MDA), xanthine oxidase (XO), 4‐hydroxynonenal (4‐HNT), superoxide dismutase (SOD) and glutathione peroxidase activity (GSH‐PX), respectively; n = 6 per group, mean ± SEM, *P < .05 vs the sham group, ^#^ *P* < .05 vs the gut IR group. Caco‐2 cells were exposed to hypoxia for 90 min, and 10 nmol/L irisin was added at the beginning of reoxygenation. (H,I) DHE fluorescence staining; (J,K) Fluo‐4 AM staining of Ca^2+^; (L,M) Western blot analysis of IRE1 and CHOP expression at 4 h after reoxygenation in Caco‐2 cells; n = 3 per group, mean ± SEM, *P < .05 vs the sham group, ^#^ *P* < .05 vs the H/R group

DHE staining of gut ROS showed that IR‐treated mice had stronger fluorescence intensity compared with the sham‐treated mice, whereas irisin significantly decreased the ROS accumulation after gut IR (Figure 3A,B). Meanwhile, irisin markedly decreased the levels of MDA, XO and 4‐HNT, while increased the levels of SOD and GSH‐Px in intestine tissues after gut IR (Figure 3C‐G). Meanwhile, neutralizing antibody against irisin significantly aggravated oxidative stress after gut IR (Figure 3A‐G). Similarly, in vitro study exhibited that irisin treatment largely reduced ROS production in Caco‐2 cells after H/R treatment (Figure 3H,I). Fluo‐4 AM was used to detect cytoplasmic calcium ions. The results revealed that irisin dramatically reduced the calcium influx induced by H/R treatment in Caco‐2 cells (Figure 3J,K). Furthermore, WB analysis manifested that irisin reversed the up‐regulation of IRE1 and CHOP expression in intestine tissues after gut IR (Figure 3L,M).

### 3.4 Irisin protects mitochondrial function to increase intercellular tight junctions and reduce enterocyte apoptosis

**Figure 4:**
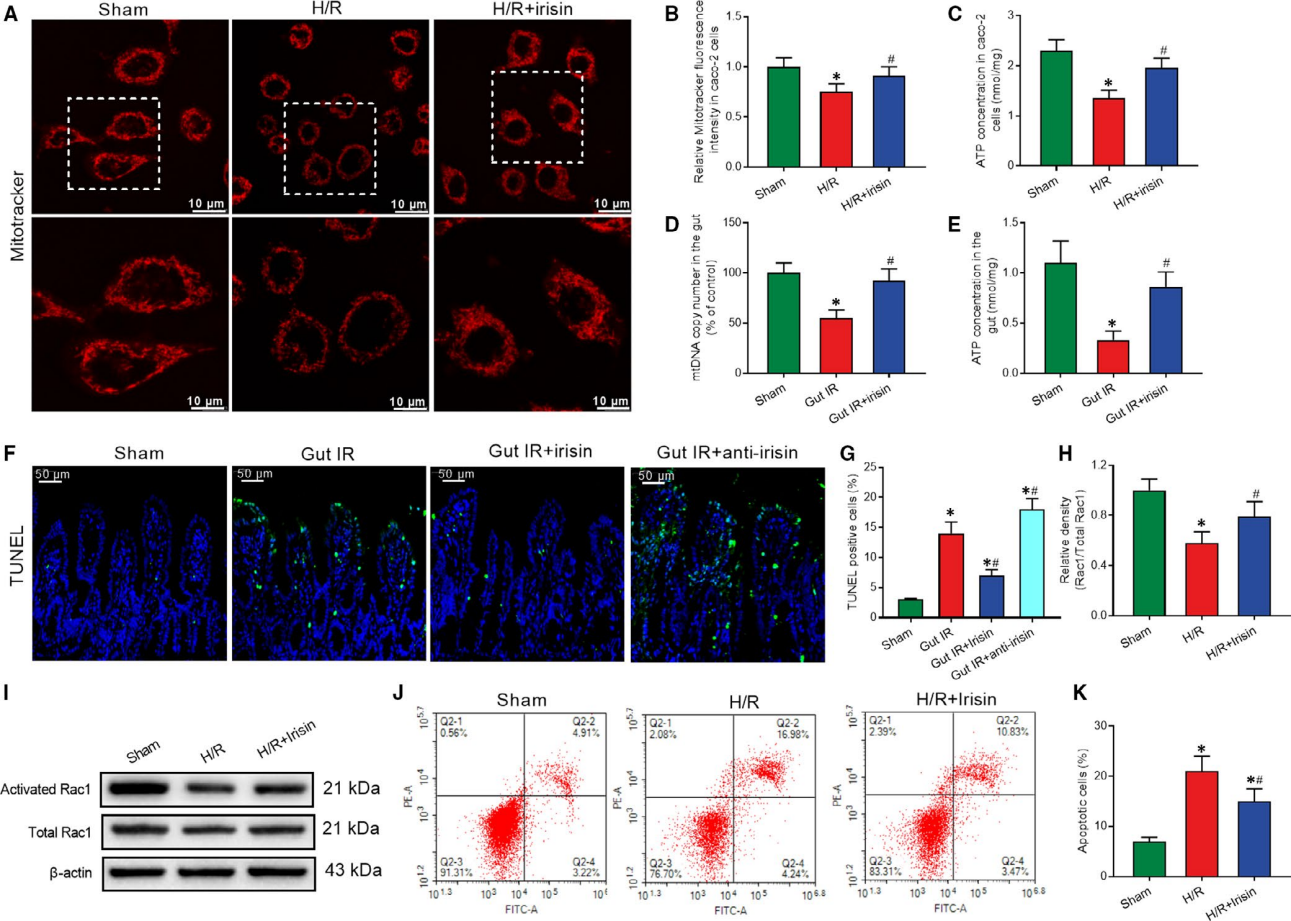
Irisin protects mitochondrial function to increase intercellular tight junctions and reduce enterocyte apoptosis. Caco‐2 cells were exposed to hypoxia for 90 min, and 10 nmol/L irisin was added at the beginning of reoxygenation. A,B, MitoTracker Red fluorescence staining of mitochondria; (C) ATP concentration at 4 h after reoxygenation in Caco‐2 cells; n = 3 per group, mean ± SEM, *P < .05 vs the sham group, ^#^ *P* < .05 vs the H/R group. Irisin (250 μg/kg in 0.5 mL saline, a single dose, iv) was administered immediately after reperfusion. Anti‐irisin (4 mg/kg; Abcam) blocking antibodies were administered at 24 h before gut IR. Four hours after reperfusion, mice were sacrificed, and tissue samples were collected. (D) Gut mtDNA copy numbers; (E) gut ATP concentration; （F,G） TUNEL fluorescence staining (green) and corresponding nuclear counterstaining (blue); n = 6 per group, mean ± SEM, *P < .05 vs the sham group, ^#^ *P* < .05 vs the gut IR group. (H,I) Western blot analysis of activation of Rac1; and （J,K） Flow cytometry analysis of apoptotic cells at 4 h after reoxygenation in Caco‐2 cells; n = 3 per group, mean ± SEM, *P < .05 vs the sham group, ^#^ *P* < .05 vs the H/R group

Mitochondrial dysfunction is the key cause of cell apoptosis. MitoTracker Red staining was conducted to determine the number of mitochondria. MitoTracker distribution and fluorescence intensity were markedly decreased after H/R treatment. Irisin treatment significantly reversed the changes in mitochondrial visualization (Figure 4A,B). Meanwhile, we found that irisin administration markedly increased the ATP concentration after H/R treatment in Caco‐2 cells (Figure 4C). mtDNA copy number in intestine tissues was detected to assess the number of mitochondria. Consistent with the in vitro results, irisin‐treated mice had higher mtDNA copy number and ATP concentration compared with the saline‐treated mice after gut IR (Figure 4D,E).

TUNEL staining was conducted to assess apoptosis of enterocytes. Mice that underwent gut IR exhibited a large number of apoptotic cells. Treatment with exogenous irisin significantly reduced the percentage of apoptotic cells and anti‐irisin antibody increased the enterocyte apoptosis at 4 hours after gut IR (Figure 4F,G). Similarly, in vitro studies showed a prominent increase in activated Rac1 in irisin‐treated Caco‐2 cells (Figure 4H,I). Meanwhile, irisin decreased the percentage of apoptotic Caco‐2 cells at 4 hours after H/R treatment (Figure 4J,K).

### 3.5 Irisin protects against gut IR injury via binding to integrin αvβ5 receptor in enterocyte

**Figure 5:**
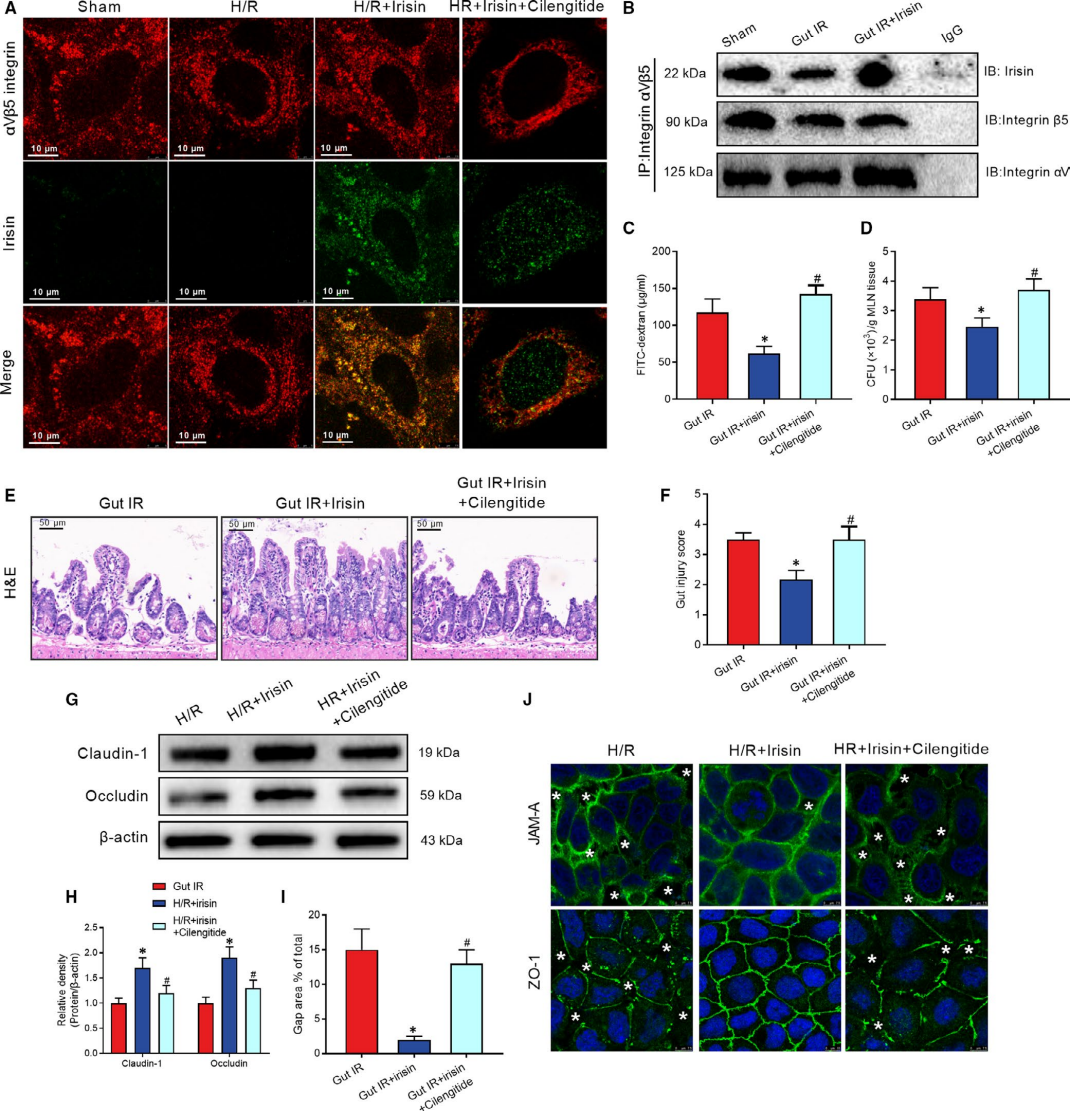
Irisin protects against gut IR injury via binding to integrin αvβ5 receptor in enterocyte. Caco‐2 cells were exposed to hypoxia for 90 min, and 10 nmol/L irisin and 20 μmol/L cilengitide trifluoroacetate were added at the beginning of reoxygenation. A, Immunofluorescence staining of irisin (green), integrin αVβ5 (red) and the corresponding nuclear counterstaining (blue) in Caco‐2 cells at 4 h after reoxygenation in Caco‐2 cells. Irisin (250 μg/kg, iv) and cilengitide trifluoroacetate (20 mg/kg, iv) were administered immediately after reperfusion. Four hours after reperfusion, mice were sacrificed, and tissue samples were collected. (B) CO‐IP of irisin and integrin αVβ5; (C) serum FITC‐dextran levels; (D) colony‐forming units (CFUs) from mesenteric lymph node (MLN) tissues; (E) haematoxylin and eosin (H&E) staining; (F) gut injury score; n = 6 per group, mean ± SEM, *P < .05 vs the gut IR group, ^#^ *P* < .05 vs the gut IR + irisin group. (G,H) Western blot analysis of claudin‐1 and occludin expression in Caco‐2 cells; and (I,J) immunofluorescence staining of JAM‐1 and ZO‐1 and the corresponding nuclear counterstaining (blue). * in the figure represents intercellular gap. n = 3 per group, mean ± SEM, *P < .05 vs the sham group, ^#^ *P* < .05 vs the H/R group

Immunofluorescent staining revealed an observable co‐localization of irisin and integrin αvβ5 proteins after irisin administration in H/R treated caco‐2 cells (Figure 5A). Cilengitide trifluoroacetate, a cyclic RGD‐containing pentapeptide, is an inhibitor of integrin αvβ5. Cilengitide trifluoroacetate disrupted the co‐localization of irisin and integrin αvβ5 proteins in caco‐2 cells (Figure 5A). Meanwhile, CO‐IP of irisin and integrin αvβ5 proteins proved irisin could bind to the integrin αvβ5 receptor in gut (Figure 5B). The cilengitide‐treated mice lost therapeutic effects of irisin in decreasing serum FITC‐dextran, MLN bacterial loads and histological changes after gut IR (Figure 5C‐F). Similarly, cilengitide abolished the protective function of irisin in up‐regulating claudin‐1 and occludin expression and restoring intercellular tight junctions during gut IR (Figure 5G‐J).

### 3.6 Irisin restores gut barrier function after gut IR by the integrin αVβ5‐AMPK‐UCP 2 pathway

**Figure 6:**
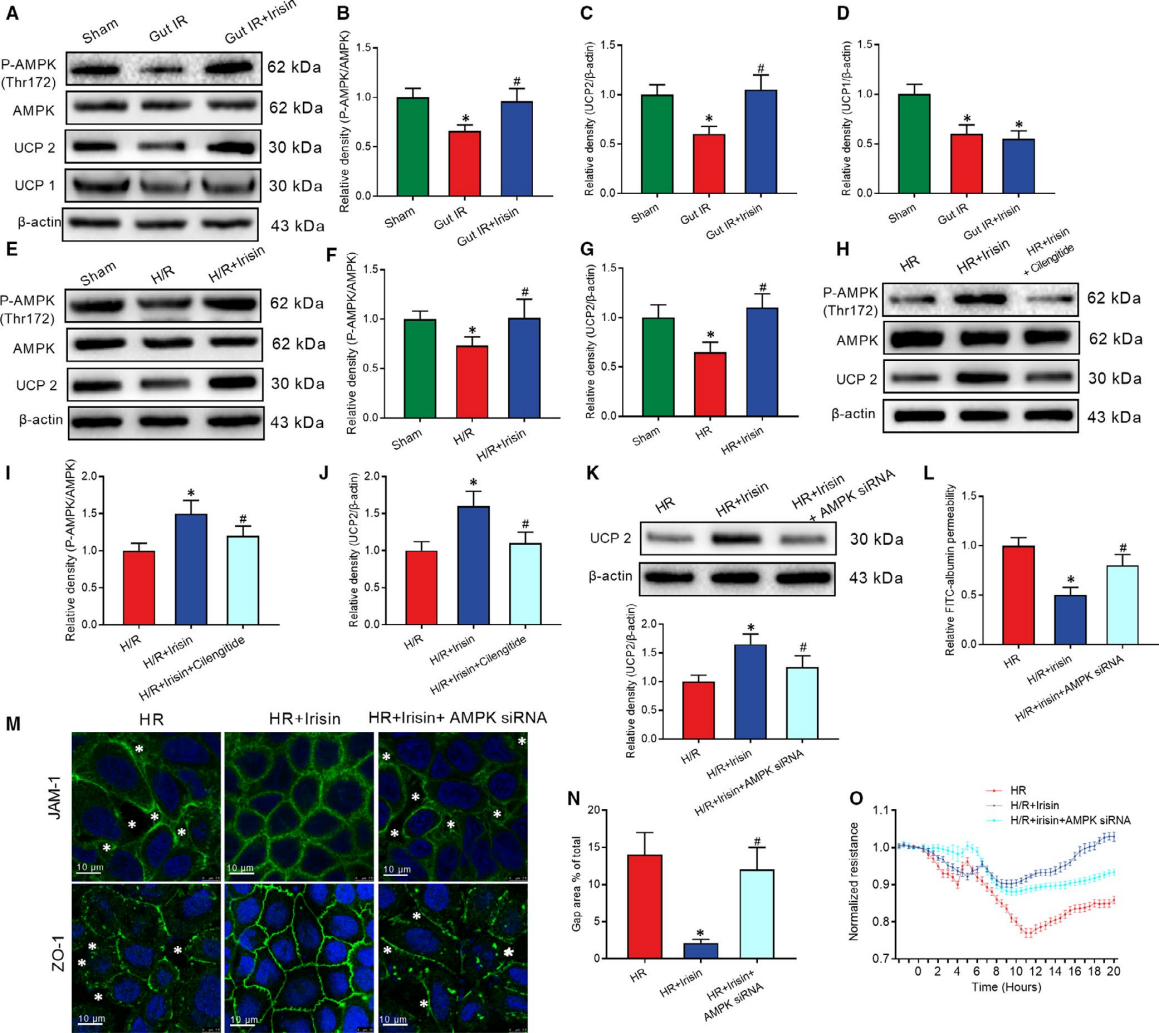
Irisin restores gut barrier function after gut IR by the integrin αVβ5‐AMPK‐UCP 2 pathway. Irisin (250 μg/kg, iv), cilengitide trifluoroacetate (20 mg/kg, iv) or genipin (20 mg/kg, iv) were administered immediately after reperfusion. Four hours after reperfusion, mice were sacrificed, and tissue samples were collected. A‐D, Western blot analysis of the activation of AMPK, UCP 2 and UCP 1 in gut tissues; n = 6 per group, mean ± SEM, *P < .05 vs the sham group, ^#^ *P* < .05 vs the gut IR group. Caco‐2 cells were exposed to hypoxia for 90 min, and 10 nmol/L irisin, 20 μmol/L cilengitide trifluoroacetate and 20 μmol/L geninpin were added at the beginning of reoxygenation. (E‐G) Western blot analysis of the activation of AMPK and UCP 2; n = 3 per group, mean ± SEM, *P < .05 vs the sham group, ^#^ *P* < .05 vs the H/R group; (H‐J) Western blot analysis of the activation of AMPK and UCP 2; (K) Western blot analysis of UCP 2 expression; (L) relative diffusion of FITC‐labelled albumin; (M) immunofluorescence staining of JAM‐1 and ZO‐1 and the corresponding nuclear counterstaining (blue). * in the figure represents intercellular gap; (N) gap area percentage; and (O) transepithelial electrical resistance (TER) at 4 h after reoxygenation in Caco‐2 cells; n = 3 per group, mean ± SEM, *P < .05 vs the H/R group, ^#^ *P* < .05 vs the H/R + irisin group

In the process of clarifying the potential mechanisms for irisin increasing intercellular tight junctions, we found that irisin dramatically replenished the decreased levels of AMPK phosphorylation at the Thr172 site and UCP 2, but not UCP 1, both in vivo and in vitro (Figure 6A‐G). Furthermore, prominent decreases in AMPK phosphorylation at the Thr172 site and UCP 2 were observed in Cilengitide‐treated Caco‐2 cells (Figure 6H‐J). To further confirm the role of AMPK in the irisin‐induced increase of intercellular tight junctions, a specific AMPK siRNA was transfected into Caco‐2 cells. UCP 2 expression was down‐regulated in Caco‐2 cells transfected with AMPK siRNA after H/R and irisin treatment (Figure 6K). Meanwhile, transfection with AMPK siRNA abolished the protective function of irisin in decreasing permeability of FITC‐albumin and gap area and increasing intercellular tight junctions and TER and after H/R treatment of Caco‐2 cells (Figure 6L‐O).

### 3.7 Genipin abolished the protective role of irisin in gut IR

**Figure 7:**
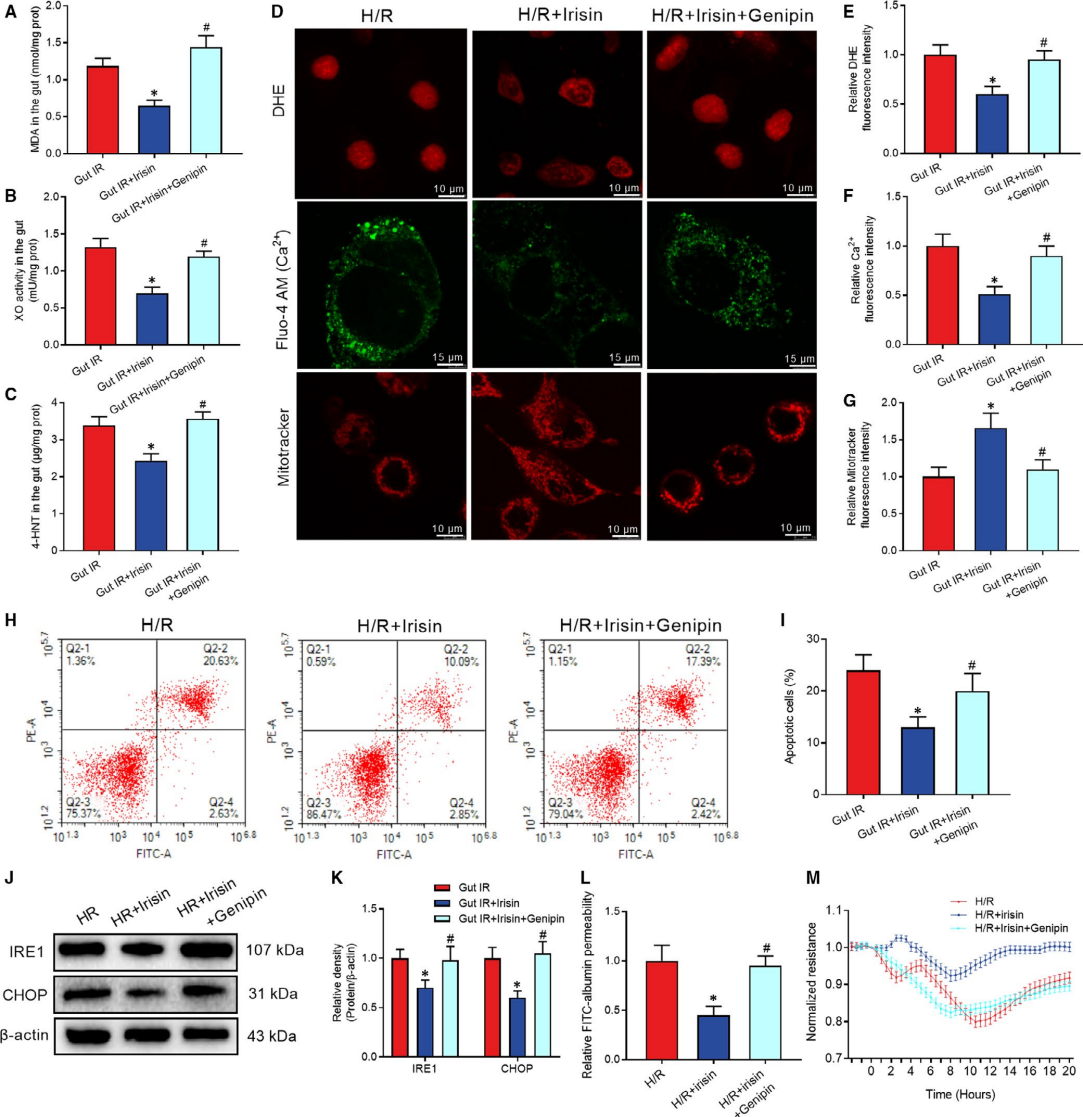
Genipin abolished the protective role of irisin in gut IR. Irisin (250 μg/kg in 0.5 mL saline, a single dose, iv) was administered immediately after reperfusion. Four hours after reperfusion, mice were sacrificed, and tissue samples were collected. A‐C, levels of gut malonaldehyde (MDA), xanthine oxidase (XO) and 4‐hydroxynonenal (4‐HNT), respectively; n = 6 per group, mean ± SEM, *P < .05 vs the gut IR group, ^#^ *P* < .05 vs the gut IR + irisin group. Caco‐2 cells were exposed to hypoxia for 90 min, and 10 nmol/L irisin was added at the beginning of reoxygenation. (D‐G) DHE fluorescence, Fluo‐4 AM and mitotracker staining; (H,I) Flow cytometry analysis of apoptotic cells at 4 h after reoxygenation in Caco‐2 cells; (J,K) Western blot analysis of IRE1 and CHOP in gut tissues; (L) relative diffusion of FITC‐labelled albumin; and (M) transepithelial electrical resistance (TER) at 4 h after reoxygenation in Caco‐2 cells; n = 3 per group, mean ± SEM, *P < .05 vs the H/R group, ^#^ *P* < .05 vs the H/R + irisin group

Genipin, a UCP 2 inhibitor, was used to clarify the role of UCP 2 in irisin‐mediated reduction of oxidative stress, calcium influx and endoplasmic reticulum. Mice received genipin showed higher levels of MDA, XO and 4‐HNT in gut tissues after irisin and IR treatment (Figure 7A‐C). Meanwhile, immunofluorescence results showed genipin abolished the protective role of irisin in decreases of ROS, calcium influx and increase of number of mitochondria in H/R treated Caco‐2 cells (Figure 7D‐G). Additionally, flow cytometry analysis showed genipin increased the percentage of apoptotic Caco‐2 cells after H/R and irisin treatment (Figure 7H,I). Moreover, genipin abolished the protective function of irisin in reduction of ER stress and permeability of FITC‐albumin and increase of TER after H/R treatment of Caco‐2 cells (Figure 7J‐M).

## 4 DISCUSSION

**Figure 8:**
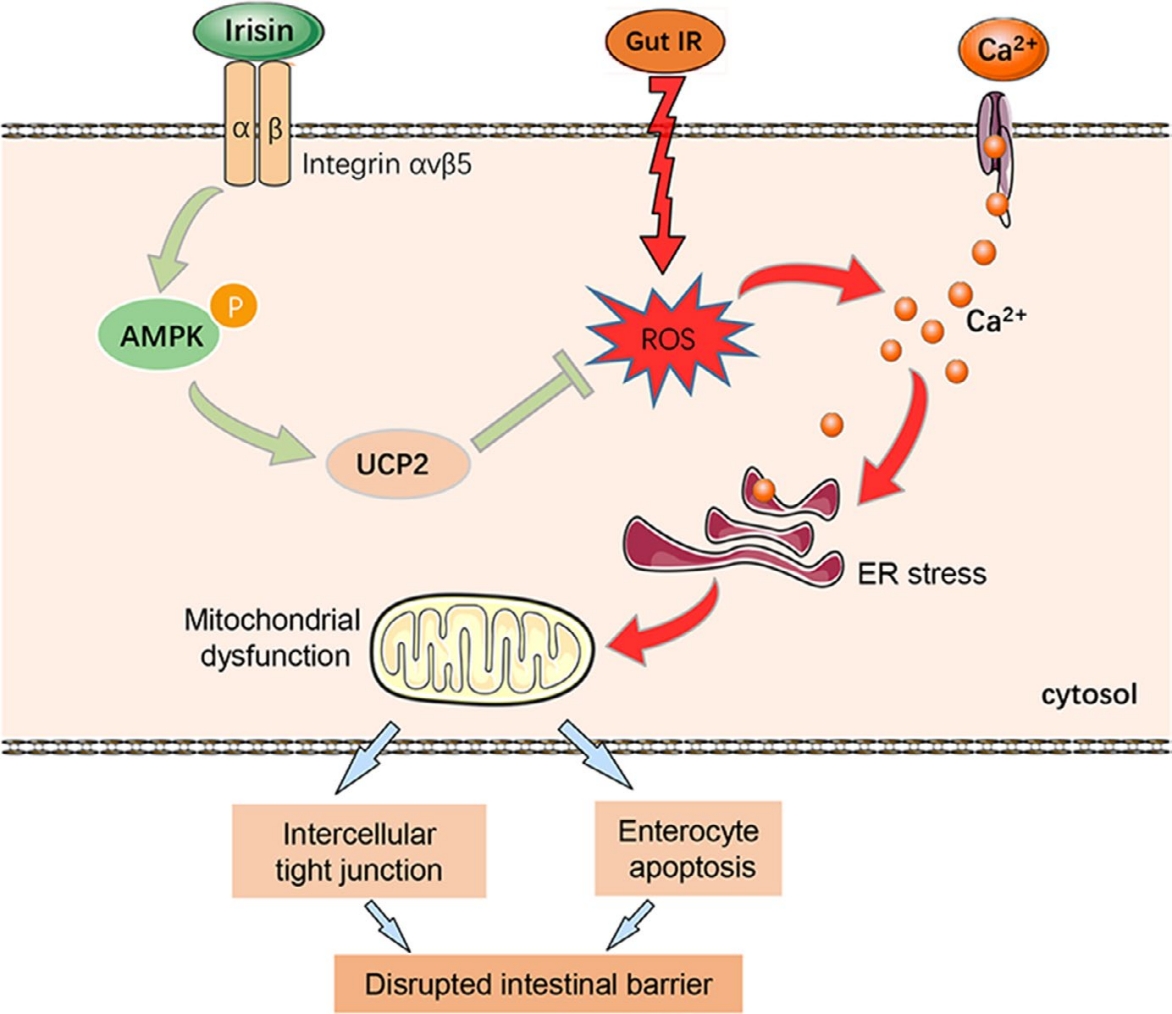
The exercise hormone irisin protects against gut barrier function after gut IR via relieving oxidative stress, calcium influx, ER stress and mitochondrial dysfunction. The potential mechanism is that irisin activates the AMPK‐UCP 2 pathway via binding to integrin αVβ5 receptor in enterocytes

In this study, we found that irisin restores gut barrier function after gut IR via relieving oxidative stress, calcium influx, ER stress and mitochondrial dysfunction. The potential mechanism is that irisin activates the AMPK‐UCP 2 pathway via binding to integrin αVβ5 receptor in enterocyte (Figure 8). Irisin therefore exhibits promising practical application prospects to solve gut barrier dysfunction‐related diseases in the future.

It has been thoroughly proven that physical exercise, mainly skeletal muscle activity, benefits the whole body, especially organs such as the heart, lung, brain and gut.^28^ Secreted irisin is derived from fibronectin type III domain containing 5 (FNDC5) protein, mainly in skeletal muscle during exercise.^14^, ^29^ The discovery of irisin fosters great expectations to clarify the mechanisms of exercise‐induced health benefits. Subsequent studies have shown that irisin is involved in obesity, cardiovascular diseases, telomere length and ageing, and hippocampal neurogenesis.^14^ Moreover, a recent study verified that irisin can directly bind to the αv class of integrin receptors in osteocytes.^19^ Previous studies have demonstrated that gut barrier dysfunction after gut IR is mainly caused by metabolic disorders and destruction of enterocytes, which can benefit from exercise.^30^, ^31^ However, the effects of irisin on the intestinal barrier have not been elucidated to date. In this study, we found that irisin protected gut barrier function via relieving oxidative stress, calcium influx, ER stress and mitochondrial dysfunction via binding to integrin αVβ5 receptor in enterocyte. Moreover, previous studies have demonstrated that macrophages play a crucial role in ischemia reperfusion injury.^32^ A recent report has confirmed the macrophages, which is a target of FNDC4, a homologue of irisin, is associated with intestinal inflammation.^33^ Therefore, it is possible that macrophages may be another target of irisin during gut IR.

The normal intestinal barrier consists of a mechanical barrier, a chemical barrier, an immune barrier and a biological barrier. The mechanical barrier is the intact intercellular tight junctions formed by enterocytes, which is the most important intestinal mucosal barrier.^34^ Intercellular tight junctions of enterocyte are consisted of tight junction proteins such as claudins, occludin, JAM and ZO‐1. The destruction of the intestinal barrier results in intraluminal microorganisms, endotoxin and other toxins entering the blood and other organs. Its destruction induces severe clinical outcomes such as sepsis, ARDS and multiple organ failure with high mortality.^3^, ^35^

Mitochondrial dysfunction is one of the main mechanisms of ischaemia‐reperfusion injury. A decrease in mitochondrial and ATP contents results in cellular energy stress and apoptosis.^36^ As an energy sensor, AMPK regulates energy metabolism via its phosphorylation and maintains cellular mitochondrial and ATP homeostasis.^13^ Moreover, previous studies proved that AMPK can directly strengthen the aggregation of cytoskeletal proteins and intercellular tight junctions via activating Rac1.^37^ Rac1 is a member of the GTPase family. Activated Rac1 (GTP‐bound state) maintains the integrity of intercellular tight junctions in the epithelial monolayer by the formation of cortical actin.^38^ As described above, irisin plays a pivotal role in energy metabolism and mitochondrial function, but whether irisin facilitates AMPK‐dependent mitochondrial protection and intercellular tight junctions has not been previously elucidated. In this study, we showed that irisin increased AMPK and Rac1 activation and relieved mitochondrial dysfunction and enterocyte apoptosis. AMPK siRNA abolished the protective effects of irisin on gut barrier function. Irisin therefore might restore gut barrier function via activation of AMPK during gut IR injury.

Enterocyte apoptosis is a key mechanism of gut barrier dysfunction.^39^ Gut IR facilitates ROS generation and eventually exceeds the antioxidant capacity of enterocytes.^40^ Excessive ROS accumulation‐induced oxidative stress reactions finally result in calcium influx, ER stress and mitochondrial dysfunction and eventual cell death.^41^ A previous study indicated that ROS scavenging is an effective method to improve cell survival.^42^ As a protective mechanism, mitochondrial UCP 1 and UCP 2 serve as a negative feedback regulator in the presence of excessive ROS. Overexpression of UCP 2 significantly decreased oxidative stress and cell apoptosis.^8^ Interestingly, irisin was proven to have an antioxidant capacity in multiple diseases.^14^ In this study, we found that irisin decreased ROS accumulation as well as increased UCP 2 expression, while there was no difference in UCP 1 expression. Inhibition of UCP 2 by genipin abolishes the protective role of irisin in gut IR. Irisin might relieve oxidative stress and enterocyte apoptosis and by up‐regulating the AMPK‐UCP 2 pathway in gut IR.

This study has some limitations. First of all, this study mainly clarified the therapeutic implications with the administration of irisin 24 hours prior to gut ischemia reperfusion injury and the additional pre‐clinical studies with post‐treatment of irisin are needed in the future. Furthermore, our study focused on the effects of irisin on the enterocyte barrier. The roles of irisin in the permeability of intestinal lymphatic vessels and blood vessels and other mechanisms of gut barrier dysfunction need further exploration. What's more, the impressive therapeutic effects of exogenous irisin on the gut IR‐induced gut barrier dysfunction were only based on basic experiments, and prospective clinical studies are needed.

In conclusion, exogenous irisin restores gut barrier function after gut IR via integrin αVβ5‐AMPK‐UCP 2 pathway. Irisin therefore exhibits promising practical application prospects to solve gut barrier dysfunction‐related diseases in the future.

## CONFLICTS OF INTEREST

We declare that there are no competing interests.

## AUTHORS’ CONTRIBUTIONS

Bi J and Zhang J participated in the research design, performed most experiments, statistical analysis and paper writing; Du Z, Wang T, Wang M and Zhang L participated in the animal studies and Western blot analysis; Ren Y and Li T participated in the cell culture and immunofluorescence. Wu Z and lv Y assisted with the design of the study. Wu R designed and supervised the study and revised the manuscript.

## Data Availability

The data are available from the corresponding author on reasonable request.
